# Biochemical Stimulus-Based Strategies for Meniscus Tissue Engineering and Regeneration

**DOI:** 10.1155/2018/8472309

**Published:** 2018-01-17

**Authors:** Mingxue Chen, Weimin Guo, Shunag Gao, Chunxiang Hao, Shi Shen, Zengzeng Zhang, Zhenyong Wang, Zehao Wang, Xu Li, Xiaoguang Jing, Xueliang Zhang, Zhiguo Yuan, Mingjie Wang, Yu Zhang, Jiang Peng, Aiyuan Wang, Yu Wang, Xiang Sui, Shuyun Liu, Quanyi Guo

**Affiliations:** ^1^Institute of Orthopedics, Chinese PLA General Hospital, Beijing Key Lab of Regenerative Medicine in Orthopedics, Key Laboratory of Musculoskeletal Trauma & War Injuries, PLA, No. 28 Fuxing Road, Haidian District, Beijing 100853, China; ^2^Center for Biomaterial and Tissue Engineering, Academy for Advanced Interdisciplinary Studies, No. 5 Yiheyuan Road, Haidian District, Peking University, Beijing 100871, China; ^3^Institute of Anesthesiology, Chinese PLA General Hospital, No. 28 Fuxing Road, Haidian District, Beijing 100853, China; ^4^Department of Bone and Joint Surgery, The Affiliated Hospital of Southwest Medical University, No. 25 Taiping Road, Luzhou 646000, China; ^5^First Department of Orthopedics, First Affiliated Hospital of Jiamusi University, No. 348 Dexiang Road, Xiangyang District, Jiamusi 154002, China; ^6^School of Medicine, Nankai University, Tianjin 300071, China; ^7^Shanxi Traditional Chinese Hospital, No. 46 Binzhou West Street, Yingze District, Taiyuan 030001, China

## Abstract

Meniscus injuries are very common and still pose a challenge for the orthopedic surgeon. Meniscus injuries in the inner two-thirds of the meniscus remain incurable. Tissue-engineered meniscus strategies seem to offer a new approach for treating meniscus injuries with a combination of seed cells, scaffolds, and biochemical or biomechanical stimulation. Cell- or scaffold-based strategies play a pivotal role in meniscus regeneration. Similarly, biochemical and biomechanical stimulation are also important. Seed cells and scaffolds can be used to construct a tissue-engineered tissue; however, stimulation to enhance tissue maturation and remodeling is still needed. Such stimulation can be biomechanical or biochemical, but this review focuses only on biochemical stimulation. Growth factors (GFs) are one of the most important forms of biochemical stimulation. Frequently used GFs always play a critical role in normal limb development and growth. Further understanding of the functional mechanism of GFs will help scientists to design the best therapy strategies. In this review, we summarize some of the most important GFs in tissue-engineered menisci, as well as other types of biological stimulation.

## 1. Introduction

Meniscus injuries are very common in athletes and middle-aged and older people [1]. The blood supply and nerve distribution to the meniscus are variable. The meniscus can be subdivided into three areas: the inner white region, which lacks a blood supply; the outer red region, which has a blood supply; and the middle red–white region, which shows transitional features. Meniscus injuries to the inner white region or middle red–white region remain hard to repair [2]. Orthopedic surgeons usually use a partial meniscectomy to treat these meniscus injuries. However, this inevitably leads to osteoarthritis (OA) of the injured knee [3]. Meniscus allograft transplantation can overcome this dilemma to an extent. However, there are some limitations to transplantation, such as viral transmission, graft preservation, and mismatching [4].

The development of tissue engineering and regeneration medicine provides a new avenue for meniscus repair. By combining cells and scaffolds, we can form tissue-engineered constructs. However, it is difficult to use these constructs to repair the injury tissues. Biomechanical or biochemical stimulation can enhance the maturation and remodeling of these constructs. Hence, we usually regard the seed cells, scaffolds, and biomechanical and biochemical stimulation as the three indispensable elements of tissue engineering. Meniscal fibrochondrocytes and stem cells are the two most important kinds of seed cell [5]. They all play critical roles in meniscus regeneration. Scaffolds can be roughly divided into scaffolds derived from synthetic polymers or from biological materials [5]. It is very hard to regenerate the injured tissue solely with a combination of seed cells and scaffold. However, biomechanical and biochemical stimulation can build a bridge between the tissue-engineered construct and the functional tissue. The biomechanical stimulation usually mimics the native meniscus biomechanical microenvironment, such as the compressive loading or tensile strength [6, 7]. However, this review focuses on the effects of biochemical stimulation of the tissue-engineered meniscus.

The most familiar biochemical stimulation is growth factor (GF). The GFs used usually play a significant role in normal limb development and growth [8–11]. They can influence cell migration, proliferation, differentiation, and apoptosis. When used for tissue regeneration, they may also play important roles in tissue maturation and remodeling. Finally, we summarize other forms of biological stimulation that are used in the tissue-engineered meniscus.

## 2. Growth Factors and Gene Therapy for Meniscus Tissue Engineering

The desirable properties and functions of the native meniscus are largely dependent on the maintenance of the unique extracellular matrix (ECM) and its structure, which is generally modulated by the anabolic and catabolic activities of meniscal cells [74]. Growing evidence indicates that in addition to genetic factors, growth factors play a key role in the metabolic activity of fibrochondrocytes and further affect development, homeostasis, and regeneration [75–78]. By binding to specific receptors on the target cell surface, growth factors may initiate signal transduction cascades and further affect cellular processes and metabolic activity. Growth factors may promote meniscus repair and regeneration via multiple mechanisms, including recruitment of fibrochondrogenic cells, enhancement of fibrochondrogenic cell proliferation, and stimulation of ECM production. Thus, local administration of growth factors may create a favorable microenvironment and further promote meniscus repair. Growth factors commonly used for meniscus and cartilage regeneration are summarized in Table 1. Of the numerous bioactive molecules, the most important and thoroughly studied growth factors include the transforming growth factor-*β* (TGF-*β*) superfamily, basic fibroblast growth factor (bFGF), and insulin-like growth factor-1 (IGF-1). The effects of other growth factors, such as connective tissue growth factor (CTGF), vascular endothelial growth factor (VEGF), platelet-derived growth factor (PDGF), hepatocyte growth factor (HGF), and platelet-rich plasma (PRP), on meniscus regeneration have also been evaluated.

### 2.1. The TGF-*β* Superfamily

The TGF-*β* superfamily consists of more than 30 members and includes TGF-*β*s, activins, and bone morphogenetic proteins (BMPs) [79, 80]. Growth factors from the TGF-*β* superfamily are involved in regulating various cellular processes, including cell survival, growth, proliferation, migration, differentiation, and apoptosis, as well as synthesis and degradation of the ECM [80–82]. TGF-*β*s and BMPs have been studied most extensively and have shown great potential in the field of tissue engineering and regenerative medicine over the past few decades [77]. Thus, it is possible that local administration of these factors may benefit functional meniscus regeneration.

#### 2.1.1. TGF-*β*s

There are three different isoforms of TGF-*β* in mammals: TGF-*β*1, TGF-*β*2, and TGF-*β*3 [83]. The most thoroughly studied TGF-*β*s in the musculoskeletal system are TGF-*β*1 and TGF-*β*3 [77]. TGF-*β* is very effective for stimulating collagen production and glycosaminoglycan (GAG) synthesis by meniscal cells [12, 14, 18, 19]. For example, Pangborn and Athanasiou [13, 15] evaluated the effects of four common growth factors (TGF-*β*1, PDGF-AB, IGF-I, and bFGF) on meniscal fibrochondrocyte ECM synthesis in monolayers or in three-dimensional (3D) cultures. These studies found that treatment with TGF-*β*1 led to the greatest amount of collagen and GAG production compared to other growth factors. Similarly, Imler et al. [16] demonstrated that TGF-*β*1 was the most potent stimulator of both collagen and GAG synthesis. In addition, a combination of TGF-*β*1 and chondroitinase ABC (C-ABC) improved the biochemical and biomechanical properties of an agarose scaffold seeded with bovine meniscus cells and articular chondrocytes [17, 18]. TGF-*β*1 also markedly increased the amount of alpha-smooth muscle actin (*α*-SMA) in meniscal cells, which plays a crucial role in the contraction of the collagen–GAG matrix [20]. Not only does TGF-*β* enhance ECM synthesis, but it also blocks matrix degradation via downregulation of proteases such as matrix metalloproteases (MMPs) or upregulation of inhibitors of MMPs (TIMPs) [21, 22]. In addition, TGF-*β* is a chief anticatabolic agent counteracting the deleterious effects of catabolic cytokines. For example, several studies [21, 22, 84] have reported that TGF-*β* may effectively suppress the catabolic effects mediated by interleukin (IL)-1 and tumor necrosis factor (TNF)-*α*, including the upregulation of MMPs (MMP-1, MMP-3, MMP-8, MMP-13, and MMP-14) and downregulation of ECM-related genes. In addition, TGF-*β* may increase TIMP production [21].

In addition to regulating matrix metabolism, another chief function of TGF-*β* in meniscus tissue engineering is the recruitment, proliferation, and fibrochondrogenic differentiation of mesenchymal stem cells (MSCs) [23, 24, 85]. In general, TGF-*β* plays a crucial role in chondrogenesis [25, 86], and studies have revealed that TGF-*β*3 has the strongest chondrogenic effects of all isoforms [26, 87, 88]. The synergistic effects of TGF-*β* and IGF-1 on the induction of dedifferentiated articular chondrocytes from their original phenotype were also observed [27]. Kulyk et al. revealed that, in this process, TGF-*β* upregulates transcription factor SOX9 through the Smad pathway, followed by enhancement of cartilage gene expression, such as type II collagen and aggrecan.

Furthermore, several studies have demonstrated that TGF-*β* supplementation can promote cartilage and meniscus repair and has promise for meniscus regeneration [28–30]. For example, Lee et al. [89] investigated the potential of anatomically correcting TGF-*β*3-infused bioscaffolds for articular cartilage regeneration. Histological and mechanical results showed that TGF-*β*3-infused bioscaffolds promoted hyaline cartilage formation in the articular surface with excellent mechanical properties similar to those of native articular cartilage 4 months after implantation in a rabbit model. Moreover, this study was the first to demonstrate that TGF-*β*3 stimulates articular cartilage regeneration through the recruitment of endogenous stem or progenitor cells, chondrogenic differentiation, and histogenesis and provided evidence that complex tissues may regenerate by homing of endogenous cells without cell transplantation. Similarly, McNulty and Guilak [90] found that applying TGF-*β*1 enhanced cellular accumulation and increased the shear strength of the repaired tissue, which suggests that TGF-*β*1 is essential for cartilage integrity and may be a potent alternative for enhancing meniscal repair.

In general, TGF-*β* is likely to push cells toward a more chondrocytic phenotype and to induce hyaline cartilage formation. Freymann et al. [91] revealed that TGF-*β* enhanced the production of specific cartilage and collagen type II proteoglycans by mesenchymal or meniscus cells but did not significantly increase the formation of type I collagen in meniscus 3D micromass or scaffold cultures, which is the main component of fibrocartilage tissue and plays a crucial role in the tensile strength of the native meniscus. Because the unique inhomogeneous feature of the meniscus presents a tremendous challenge for total meniscus regeneration, the use of various combinations of growth factors may be a promising solution. Lee et al. [31] spatiotemporally delivered a combination of CTGF and TGF-*β*3 to regenerate the meniscus in a sheep model. They demonstrated that spatiotemporally delivered CTGF and TGF-*β*3 could lead to heterogeneous meniscus regeneration by inducing endogenous stem/progenitor cells to differentiate and synthesize zone-specific types I and II collagen.

However, several unfavorable side effects of TGF-*β*1 treatment must be mentioned, such as induction of synovial fibroplasias and fibrosis, stimulation of osteophyte formation, and recruitment of inflammatory leukocytes [84, 92, 93]. Fortunately, studies have reported that applying local inhibitors of TGF-*β* may help block these undesirable side effects [84].

#### 2.1.2. Bone Morphogenetic Proteins (BMPs)

BMPs also belong to the TGF-*β* superfamily and play a crucial role in bone and cartilage formation and repair [94–96]. They share several functions with TGF-*β*s and have potential for meniscal regeneration [97]. The distinct and key function of BMPs is osteogenic differentiation of human MSCs (hMSCs), but they are not restricted to bone and also induce osteoblastic [98, 99], tenogenic [100], and chondrogenic [101] differentiation of MSCs. For instance, a study examined the capacity of BMP-2, BMP-4, and BMP-6 to promote chondrogenic differentiation of MSCs and encourage cartilage formation* in vitro*. It found that BMP-2 is more effective than others for chondrogenic differentiation of MSCs [39]. Gouttenoire et al. [32] reported the specific capability of BMP-2 to reverse chondrocyte dedifferentiation by increasing cartilage-specific collagen type II production in dedifferentiated chondrocytes. In addition, Shintani and Hunziker [42] evaluated the potential of BMP-2, BMP-7, and TGF-*β*1 for inducing chondrogenic differentiation of synovial explants. In that study, all three growth factors induced chondrogenic differentiation of synovial MSCs and enhanced the formation of cartilaginous tissue. However, BMP-7 was more potent and effective than the other growth factors in inducing chondrogenesis. In addition, Miyamoto et al. [41] reported that the effects of BMP-7 on chondrogenic differentiation of MSCs were enhanced when it was combined with TGF-*β*3.

BMPs have a clear role in modulating tissue homeostasis and blocking degradation processes [102, 103]. Chubinskaya compared the anabolic activity of BMPs (BMP-2, BMP-4, BMP-6, and BMP-7) and cartilage-derived morphogenetic proteins (CDMP-1 and CDMP-2) in human articular chondrocytes [40]. BMP-2, BMP-4, and BMP-7 were more potent in upregulating proteoglycan production than the other three growth factors, with the highest proteoglycan content on day 9 in the presence of BMP-7. Furthermore, under simultaneous treatment with IL-1*β* and the aforementioned growth factors, only BMP-7 effectively antagonized the inhibition of proteoglycan synthesis mediated by IL-1*β*. Similar studies have also demonstrated that BMP-7 effectively counteracted chondrocyte catabolism induced by various proinflammatory cytokines such IL-1, IL-6, IL-8, MMP-1, MMP-13, and TNF-*α* [35, 36, 67, 104]. The prominent proanabolic and anticatabolic properties of growth factors are essential to their clinical application, particularly under local inflammatory conditions triggered by trauma, degenerative disease, or surgery. In addition, BMP-7 increases matrix synthesis without stimulating uncontrolled fibroblast proliferation and osteophyte formation [105, 106]. It is interesting that BMP-2 may enhance matrix turnover in native and IL-damaged cartilage, as evidenced by upregulated collagen type II and aggrecan expression and increased aggrecan degradation, which suggests that BMP-2 treatment may lead to a reparative response in chondrocytes after cartilage injury or osteoarthritis [33].

BMPs effectively promote cartilage and osteochondral regeneration [107, 108]. BMPs have also been evaluated for their potential in meniscus repair and regeneration. For example, Forriol et al. [37] investigated the effects of BMP-7 treatment on meniscal defects in a sheep model. In that study, defects were made in the avascular region of the medial meniscus and treated with Putty® (control group) or osteogenic protein-1 (OP-1) Putty, which contains BMP-7 (experimental group). After 12 weeks, meniscal defects in the experimental group were filled with cellular fibrous tissue that connected both edges of the defects. In addition, Minehara et al. [34] developed a chemotactic cell-seeding technique with a solvent-preserved human meniscus scaffold to improve meniscal repair. They found that rhBMP-2 stimulated chondrocyte migration and proliferation as well as proteoglycan production throughout the meniscus tissue, which suggests a potential application of rhBMP-2 as a chemokinetic factor for loading into a scaffold for cartilage and meniscus tissue engineering. In another study, the Achilles tendon was treated with BMP-7 and transplanted into a rat meniscal defect model [38]. After 12 weeks, regenerated meniscus-like tissue was observed; this may effectively prevent cartilage degeneration.

In general, BMP-2 and BMP-7, which are approved by the Food and Drug Administration (FDA) for clinical use, have shown promising effects on chondrocyte differentiation, ECM production, and fibrocartilaginous tissue regeneration. Synergistic effects have been observed when BMP-7 is combined with other growth factors such as IGF-1 and TGF-*β*1 [109], which suggests that the addition of these factors may lead to greater improvements in meniscus repair and regeneration.

### 2.2. Basic Fibroblast Growth Factor (bFGF)

The fibroblast growth factor (FGF) family is composed of 18 structurally related signaling molecules [110, 111]. Basic FGF, also known as bFGF, FGF2, or FGF-*β*, is an important member of the FGF family and is found in the cartilaginous matrix [78, 102, 112, 113]. The mitogenic effects of FGF on MSCs were first reported by Oliver more than 27 years ago [114]. FGF is a powerful mitogen for a variety of cell types, including chondrocytes, fibrochondrocytes, osteoblasts, and adipocytes [102]. Numerous studies [45, 115, 116] have demonstrated the strong stimulating effects of bFGF on meniscal cell proliferation in monolayer cultures as well as in tissue-engineered constructs. For example, one study [44] evaluated the effects of nine growth factors (EGF, NGF, IGF-1, TGF-*α*, TGF-*β*, a-FGF, b-FGF, PDGF-AA, and PDGF-AB) on meniscal fibrochondrocyte proliferation in a monolayer culture. EGF, bFGF, TGF-*α*, and PDGF-AB stimulate cell proliferation, with bFGF having the greatest effect. Cucchiarini et al. [46] investigated the effects of bFGF on proliferation and metabolic activities of meniscal cells using a gene-based approach in which bFGF was vectored with a recombinant adeno-associated virus; increased cell proliferation and alpha-smooth muscle actin (*α*-SMA) expression were observed. Sotiropoulou et al. [47] also demonstrated that adding bFGF to culture media enhanced the proliferative capacity of hMSCs. In addition, FGF maintains the multilineage differentiation potential of MSCs during proliferation, including chondrogenic, osteogenic, adipogenic, and neurogenic differentiation [48, 49, 117, 118].

Monolayer expansion may result in loss of expression of collagen type II and matrix-forming phenotypes of meniscal cells. However, Adesida et al. [50] demonstrated the ability of bFGF to restore the chondrogenic phenotype of passaged meniscal cells based on reexpression of collagen type II and proteoglycan at both the gene and protein levels. In that study, supplementation with bFGF upregulated expression of collagen type II 200-fold in subsequent 3D pellet cultures. Moreover, this favorable effect was further enhanced under 5% oxygen culture conditions.

The specific role of bFGF in anabolic and catabolic processes remains controversial. Studies have demonstrated the capacity of bFGF to stimulate the anabolic activity of cartilage and meniscus [13]. For example, Tumia and Johnstone [115] demonstrated that meniscal cells from all zones responded positively to bFGF supplementation by enhancing DNA synthesis and ECM formation. Cheng et al. [119] also demonstrated that bFGF boosted the kinetics of MSC chondrogenesis, resulting in faster differentiation and leading to enhanced ECM accumulation. However, several studies [16, 51] revealed that bFGF was the least effective stimulator of both protein and proteoglycan production. In addition, Loeser et al. [54] reported that bFGF had dramatic antagonistic effects on proteoglycan accumulation promoted by IGF-1 and/or BMP-7. bFGF is also an antagonist of collagen type II and decorin production induced by IGF-1 and TGF-*β* in porcine articular chondrocytes [52]. Moreover, some evidence suggests that bFGF leads to the upregulation of MMPs and aggrecanases and increases reactive oxygen species such as nitric oxide (NO) and the superoxide anion [53]. The role of two members of the FGF family, FGF-2 and FGF-18, in cartilage homeostasis was evaluated by Ellman et al. [120, 121], who concluded that bFGF is a catabolic mediator in human cartilage via increased matrix-degrading enzyme activity and decreased ECM production, whereas FGF-18 is likely an anabolic regulator in human articular chondrocytes, enhancing ECM formation and chondrogenic differentiation and inhibiting cell proliferation.

Despite its controversial effects on cartilage homeostasis, the effects of bFGF on tissue regeneration and repair were investigated and revealed positive results [43, 55, 122, 123]. Deng et al. [55] developed gelatin microspheres in combination with controlled-release bFGF for cartilage repair in a rabbit model. Histological results showed that defects were filled with hyaline-like cartilage after 24 weeks, which indicates the great potential of a bFGF-loaded scaffold for promoting cartilage regeneration. In another study [56], meniscal cells were cultured on poly L-lactic acid (PLLA) scaffolds with bFGF under hypoxic conditions. After 4 weeks, histological results demonstrated synergic effects of hypoxia and bFGF on enhancing GAG production and compressive properties of tissue-engineered meniscus constructs* in vitro*. In addition, Ionescu et al. [57] investigated the effects of bFGF as a promitotic manager and TGF-*β*3 as a promatrix formation manager on meniscus repair and integration with electrospun polycaprolactone (PCL) scaffolds. This study biochemically, histologically, and mechanically showed that short-term delivery of bFGF or sustained delivery of TGF-*β*3 enhanced integration for bovine meniscus tissues, which suggests that both bFGF and TGF-*β*3 have the potential to promote meniscus repair.

Miyakoshi et al. [124] reported that local administration of bFGF led to inflammatory responses and osteophyte formation in a rabbit model. bFGF was also closely associated with synovial proliferation and hyperplasia in rheumatoid arthritis joints [120]. Given the potentially deleterious effects and controversial role of bFGF in articular development, the use of bFGF for meniscus repair and regeneration must be further explored. Although there is less literature on the role of FGF-18 in meniscus repair, it has shown promising anabolic effects on chondrocytes [121, 125]. More detailed studies are necessary to define the exact effects of FGF on meniscal cells, explants, and engineered constructs and to provide evidence for meniscus regeneration.

### 2.3. Insulin-Like Growth Factors (IGFs)

Two distinct forms of IGFs, IGF-1, and IGF-2 play a pivotal role in tissue metabolism [126]. IGF-1 is a vital anabolic growth factor of cartilaginous tissue under normal conditions and has been studied most in cartilage and meniscus tissue engineering [67, 127]. In general, IGF-1 enhances anabolic effects and inhibits catabolic responses. Puetzer et al. [58] explored the effects of IGF-1 on the mechanical and biochemical properties of meniscal constructs. After 4 weeks, IGF-1 treatment led to a 26-fold increase in GAG production, a 10-fold increase in collagen production, and a 3-fold increase in the equilibrium modulus of engineered meniscal constructs compared to 0-week controls, providing evidence for IGF-1 as a potential treatment in meniscal regeneration. A mixture of bFGF, TGF-*β*1, and IGF-1 elevates expression of collagen type II and aggrecans in fibroblast-like synoviocytes* in vitro* [66]. In another study [59], IGF-1 applied to monolayer cultures for 48 h enhanced fibrochondrocyte proliferation and formation of ECM in all zones of the meniscus. What is interesting is that the meniscal cells from the avascular region responded more favorably than those from the vascular region. This indicates that fibrochondrocytes from avascular meniscal tissue are able to express their intrinsic potential to regenerate when exposed to suitable growth factors. In addition, IGF-1 effectively decreases expression of aggrecanase-1 and reduces the release of degrading molecules such as MMPs [67]. For example, Im et al. [67] demonstrated the prominent suppressive effects of the combination of IGF-1 and BMP-7 on MMP-13 expression: IGF-1 and BMP-7 decreased inflammatory cytokine expression and/or their intermediate gene products. However, chondrocyte responsiveness to IGF-1 may diminish with age and under conditions of inflammation due to overexpression of IGF binding proteins [128–131]. BMP-7 seems attractive for counteracting these effects because of its robust anticatabolic actions. Therefore, synergistic effects resulting in enhanced ECM production were observed when BMP-7 and IGF-I were used in combination [109, 132].

Apart from its anabolic effects on cartilaginous tissue, IGF-1 promoted the chondrogenic differentiation of MSCs, which may be further enhanced when combined with TGF-*β*1 [60, 64]. IGF-1 also stimulated cell migration in specific regions of the meniscus [61]. Together, these findings support the regenerative potential of IGF for cartilage and meniscus tissue engineering. Zhang et al. [62] studied the ability of human IGF-1- (hIGF-1-) meshed bone marrow stromal cells (BMSCs) to promote the repair of full-thickness meniscal defects and found that defects were completely filled with white tissue 16 weeks after treatment in a goat model that was histologically and biochemically similar to normal meniscal fibrocartilage. In another study, Goodrich et al. [63] investigated the ability of chondrocytes modified by an adenovirus vector encoding equine IGF-1 to enhance cartilage healing in an equine model. Histological results showed that defects were filled with a more hyaline-like tissue at 8 months, whereas control defects were covered with irregular and more fibrous tissue. Izal et al. [65] also reported that treatment with TGF-*β*1 and IGF-1 in combination improved repair of the meniscus avascular zone by enhancing meniscal cell proliferation and tissue attachment.

### 2.4. Other Growth Factors and Small Bioactive Molecules

In addition to the aforementioned growth factors, other growth factors, such as CTGF, VEGF, PDGF, HGF, and PRP, have been studied for their ability to improve meniscal repair and regeneration. Inducing angiogenesis may be essential to enhancing the healing capacity of the meniscus tissue. Thus, VEGF seems to be attractive for improving meniscus repair by stimulating angiogenesis. However, Petersen et al. [68, 133] demonstrated that VEGF failed to promote healing in the meniscus in a sheep meniscal longitudinal injury model treated with VEGF-coated sutures. In addition, HGF induces blood vessel formation in engineered meniscal fibrochondrocyte-polyglycolic acid (PGA) constructs without increasing any mechanical properties [69]. The effects of CTGF were also assessed. He et al. [70] studied the reparative effects of CTGF on enhancing meniscal repair in the meniscal avascular zone in a rabbit model and demonstrated that CTGF might promote healing of defects by stimulating ECM deposition within the repair zone. Nishida et al. [71] also reported that CTGF-hydrogel collagen scaffolds enhanced articular cartilage regeneration in a rat model. PDGF also enhanced wound healing and ECM production as well as cell proliferation; thus, it was believed to be capable of enhancing tissue regeneration and repair [126]. Tumia and Johnstone [72] investigated the capacity of PDGF-AB to improve meniscal tissue regeneration in all three zones of the meniscus and found that it enhanced fibrochondrocyte proliferation and new matrix formation, which suggests that PDGF may be of benefit in meniscus regeneration. PRP is the source of multiple growth factors, including TGF-*β*, PDGF, VEGF, IGF-1, bFGF, and EGF [73, 134]. Because growth factors are effective in meniscal healing, PRP may be a potent agent for meniscal repair. PRP promoted cartilage regeneration by increasing ECM content in a rabbit model [134]. Ishida [73] also demonstrated the ability of PRP to encourage matrix deposition and proliferation of meniscal cells in a monolayer culture, histologically enhancing the healing of meniscal defects with tissue that resembled the inner region of the meniscus in a rabbit model. However, Zellner et al. [135] reported no improvements in the healing of a meniscus defect after treatment with PRP-loaded hyaluronan-collagen composite matrices.

Several small bioactive molecules, such as kartogenin (KGN), aptamer, Y-27632, and E7 peptide, have also attracted great interest and shown promising results in cartilage and meniscus regeneration. For example, Huang et al. [136] reported that KGN not only promoted chondrogenic differentiation of tendon stem cells* in vitro* but also enhanced meniscus-like tissue formation in a rabbit model. Hu et al. [137] demonstrated that an aptamer-bilayer scaffold could specifically recognize and bind with MSCs, while efficiently recruiting them to enrich the MSCs around the osteochondral defect, successfully achieving osteochondral regeneration. A biphasic scaffold functionalized with E7 peptide was also demonstrated to enhance cartilage regeneration via the specific homing of endogenous stem cells [138]. The rho-kinase inhibitor Y-27632 also favors the differentiation of chondroprogenitors and prevents the dedifferentiation of articular chondrocytes [139, 140].

### 2.5. Gene Therapy

Gene therapy is a novel approach to meniscus tissue engineering that aims to transfer specific genes into an organism or tissue using viral or nonviral vectors or direct injection [102, 141]. Because growth factor concentration gradients and the duration of treatment may play a crucial role in meniscus repair, an important aspect of growth factor treatment is their delivery, given their short biological half-life and rapid clearance potential. To achieve controlled and extended growth factor release, gene transfer techniques may be favored for the local administration of specific factors. The major advantage of gene therapy is high-concentration delivery and persistent expression of these growth factors at the repair site [141–144]. A variety of vectors are currently used for gene transfer in meniscus tissue engineering, including nonviral vectors, adenoviral vectors, retroviral/lentiviral vectors, herpes simplex virus (HSV) vectors, and recombinant adeno-associated virus (rAAV) vectors. Each exhibits specific characteristics [46, 62, 69, 145–149]. In general, nonviral vectors are safe because of their lack of inherent replication capability, but their lower efficiency limits their widespread application [150, 151]. Adenoviral vectors are characterized by high transduction efficiency and a low risk of carcinogenesis, but they are immunogenic and fail to maintain long-term transgene expression [147, 152, 153]. Retroviral/lentiviral vectors permit prolonged transgene expression by integrating into the host cell genome. Although retroviral vectors only transduce actively replicating cells, this obstacle can be overcome with the application of lentiviral vectors, which are most effective for transduction in nondividing cells [153–156]. However, the potential for insertional mutagenesis and tumor gene activation makes them unattractive candidates for clinical application. The advantage of HSV vectors is their ability to deliver long transgenes and a relatively high level of transduction efficiency in nondividing cells. Nevertheless, several studies have revealed that they are toxic and mediate only short-term transgene expression [154]. rAAV vectors are promising gene vehicles because they not only exhibit high transduction efficacy in both dividing and nondividing cells but also allow for long-term transgene expression [46, 148, 149, 154, 157]. In addition, these vectors demonstrate fewer immunogenic effects and are not pathogenic. All of these features make them an attractive choice for tissue engineering. Currently, there are two primary methods of gene transfer: direct injection of a gene into the target tissue and implantation of a genetically modified cell into the body. Numerous experiments have shown the feasibility of gene transfer to several tissues of the musculoskeletal system. Thus, gene transfer of regenerative factors appears to be a promising option for promoting meniscal repair and regeneration.

To date, only a few studies have used gene therapy strategies for meniscus tissue engineering. Growth factors used for gene transfer, including TGF-*β*1, HGF, and bFGF, have demonstrated the potential to enhance meniscus repair and regeneration. For example, bFGF vectored with rAAV enhanced cell proliferation, cell survival, and *α*-SMA expression in human meniscal fibrochondrocytes* in vitro*. This study demonstrated the feasibility of gene transfer for aiding in the repair of meniscal defects [158]. In another study [149], human meniscal fibrochondrocytes modified using a TGF-*β* rAAV enhanced cell proliferation and matrix synthesis. The TGF-*β* rAAV vectors were injected directly into the defects in meniscal explants and promoted repair of the meniscus lesions. In addition, meniscal cells and MSCs transduced with adenoviral vectors encoding TGF-*β*1 and seeded type I collagen–GAG matrices were transplanted into the injured avascular region of bovine menisci. After 3 weeks of* in vitro* culture, the constructs showed enhanced cellularity, collagen and proteoglycan production, and repair of the meniscal lesions with new tissue [147]. Previously, we also mentioned a study in which BMSCs with the transfected hIGF-1 gene were mixed with calcium alginate gel and aided in the repair of meniscus defects [151]. In general, gene therapy has the potential to enhance meniscus repair and regeneration, but more work is needed to identify the best candidate genes and ideal combinations of genes for meniscus regeneration.

### 2.6. Other Types of Biological Stimulation

Because a lack of blood supply creates a hypoxic environment in the inner region of the meniscus, scientists have attempted to mimic this environment in* in vitro* cultures to restore a differentiated phenotype. For example, Adesida et al. [50] demonstrated that low oxygen tension may enhance the matrix-forming phenotype of meniscal cells. Meniscal cells from the inner and outer regions responded differently to hypoxia culture. Indeed, cells from the outer meniscus showed greater sensitivity to lowered oxygen tension than cells from the inner meniscus. The response of meniscal cells to hypoxia was mediated by transcription factor hypoxia inducible factor-1*α* (HIF-1*α*), which may play a crucial role in determining the phenotype of inner meniscus cells [159]. Additive and synergistic effects of bFGF and hypoxia were also found on the enhancement of GAG accumulation and the compressive properties of engineered meniscus constructs* in vitro* [56].

Coculture systems have also been used for meniscus tissue engineering. Gunja and Athanasiou [160] used cocultures of second-passage meniscus cells and primary articular chondrocytes at varying ratios (100 : 0, 75 : 25, 50 : 50, 25 : 75, and 0 : 100) to enhance the biochemical and biomechanical properties of engineered constructs. Coculture systems with a higher percentage of chondrocytes led to significantly greater production of collagen type I, collagen type II, and GAG as well as compressive properties, whereas constructs with a higher percentage of meniscus cells resulted in a greater collagen type I content. This study illustrates that it is possible to achieve matrix content and mechanical properties close to native values of the meniscal inner and outer regions using a variety of coculture ratios. In another study, Cui et al. [161] investigated the use of coculture systems of human meniscal cells with MSCs at different ratios (100 : 0, 75 : 25, 50 : 50, 25 : 75, and 0 : 100) for meniscus tissue engineering and regeneration. The 75% meniscal cell/25% MSC coculture system showed optimal meniscus ECM production and the lowest hypertrophic expression of MSC genes such as COL10A1 and MMP13 during chondrogenic differentiation. This study indicates that coculture of meniscal cells with MSCs has the potential to expand the limited supply of meniscal cells with enhanced ECM production without hypertrophy.

## 3. Conclusion, Challenges, and Future Perspectives

Currently, there is no ideal treatment for the meniscus injuries. Tissue engineering is an attractive and promising strategy for repairing or regenerating the meniscus defects. However, regeneration of the functionally engineered meniscus remains challenging because of its complicated structure and functions. This review clearly demonstrates that biochemical stimulus plays a crucial role in the repair and regeneration of the engineered meniscus. There seems to be great potential for growth factors loading to promote regeneration of the engineered meniscus.

Individual growth factors are potent stimulators and have significant effects on many cellular processes, such as cell proliferation, differentiation, and metabolic activity, as well as meniscus repair and regeneration. Numerous experimental studies have demonstrated the synergistic effects of combinations of growth factors on meniscus repair and regeneration. Future investigations are warranted to explore the effects on meniscus tissue engineering of small bioactive molecules, which have already been shown to promote cartilage regeneration. The design of a good growth factor application strategy needs to consider the spatiotemporal specificity of meniscus regeneration. Nevertheless, it is easier than ever to achieve a spatial-specific growth factors distribution using a 3D printing approach. It is also important to develop a sequential release model for multiple growth factors that mimics cell migration and proliferation in the early regeneration stage, differentiation in the middle regeneration stage, and tissue remodeling in the final maturation stage. Gene therapy approaches may help to achieve this in the future.

Physical mechanical loading also plays a vital role in the development, remolding, and regeneration of the meniscus. The combination of mechanical stimulation and growth factors may yield great benefits for meniscus tissue engineering in the future.

## Figures and Tables

**Table 1:** Commonly used growth factors for cartilage and meniscus tissue engineering and regeneration.

Growth factor	Cell types	Culture conditions	Findings	Authors and reference
TGF-*β*1	Meniscal fibrochondrocytes	Monolayer culture	Increase collagen and GAG synthesis	Tanaka et al. [12]
				Pangborn and Athanasiou [13]

TGF-*β*	Meniscal fibrochondrocytes	Monolayer/alginate beads/explant culture	Increase proteoglycan synthesis	Collier and Ghosh [14]

TGF-*β*1	Meniscal fibrochondrocytes	PGA scaffold culture	Increase collagen and GAG synthesis	Pangborn and Athanasiou [15]

TGF-*β*1	Meniscal fibrochondrocytes	Meniscus explant culture	Increase collagen and GAG synthesis	Imler et al. [16]

TGF-*β*1 and C-ABC	Cocultures of meniscal fibrochondrocytes and articular chondrocytes	Agarose scaffold culture	Increase collagen synthesis and the Young's modulus and ultimate tensile strength	MacBarb et al. [17]

TGF-*β*1 and C-ABC	Meniscal fibrochondrocytes and articular chondrocytes	Agarose scaffold culture	Enhance compressive and tensile properties	Huey and Athanasiou [18]

TGF-*β*1 and HP	Meniscal fibrochondrocytes	PLLA scaffold culture	Increase collagen and GAG deposition and compressive properties	Gunja et al. [19]

TGF-*β*1	Meniscal fibrochondrocytes and articular chondrocyte	Monolayer culture	Increase SMA content	Zaleskas et al. [20]

TGF-*β*1	Articular chondrocytes	Explant culture	Reduce MMP-1, MMP-3, MMP-8, and MMP-13 expression and induced TIMP-2 and TIMP-3 production	Hui et al. [21]

TGF-*β*1	Articular chondrocytes	Monolayer culture	Suppress MMP-13, MMP-14 expression	Takahashi et al. [22]

TGF-*β*1	Human synovium-derived stem cells	Monolayer culture	Enhance chondrogenic differentiation and proliferation	Kim et al. [23]

TGF-*β*1	BMSCs	Monolayer culture	Enhances chondrogenic differentiation and proliferation	Jian et al. [24]

TGF-*β*	MSCs	Monolayer culture	Induce chondrogenic differentiation	Augustyniak et al. [25]—
				Tang et al. [26]

TGF-*β*1, TGF-*β*2, IGF-I	Dedifferentiated adult human articular chondrocytes	Monolayer culture	Reexpress aggrecan and type II collagen genes	Yaeger et al. [27]

TGF-*β*1	BMSCs	Chitosan/gelatin scaffolds culture	Promoted chondrogenic differentiation of MSCs, cartilage matrix synthesis, repair of rabbit cartilage defects	Diao et al. [28]

TGF-*β*3	BMSCs	PLGA-gelatin/chondroitin sulfate/hyaluronic acid hybrid scaffold in rabbit	Enhance MSCs proliferation and abundant ECM production, cartilage regeneration	Fan et al. [29]

TGF*β*3	None	PCL-HA scaffold in rabbit	Regenerate articular cartilage by homing of endogenous cells	Bochyńska et al. [30]

TGF*β*3 and CTGF	None	PCL scaffold in sheep	Lead to heterogeneous meniscus regeneration	Lee et al. [31]

BMP-2	Dedifferentiated articular chondrocytes	Monolayer culture	Reverse chondrocyte dedifferentiation	Gouttenoire et al. [32]

BMP-2	None	Intra-articular injection in mice	Enhance matrix turnover in native and IL-damaged cartilage	Davidson et al. [33]

BMP-2	Articular chondrocytes	Solvent preserved human meniscus explant culture	Stimulate chondrocytes migration and proliferation and enhance meniscus repair	Minehara et al. [34]

BMP-7	Articular chondrocytes	Monolayer culture	Counteract chondrocyte catabolism induced by proinflammatory cytokines	Elshaier et al. [35]
				Huch et al. [36]

BMP-7	None	Intra-articular injection in sheep	Fill meniscal defect with cellular fibrous tissue	Forriol et al. [37]

BMP-7	None	Injection into Achilles tendon in rats	Regenerate meniscus-like tissue	Ozeki et al. [38]

BMP-2/-4/-6	hBMSC	Monolayer culture	Enhance chondrogenic differentiation	Sekiya et al. [39]

BMP-2/-4/-6/ -7 and CDMP-1/-2	Articular chondrocytes	Alginate beads culture	BMP-7 are most potent in upregulating proteoglycan production and counteract catabolic activity mediated by IL-1	Chubinskaya et al. [40]

BMP-7 and TGF-*β*1	Synovial mesenchymal stem cells	Pellet culture	Enhance chondrogenesis from synovium-derived MSCs	Miyamoto et al. [41]

BMP-2/BMP-7, TGF-*β*1	Synovial tissue	Agarose scaffold culture	Enhance chondrogenic differentiation of synovial explants	Shintani and Hunziker [42]

bFGF	Meniscal fibrochondrocytes	Monolayer culture	Enhance proliferation	Hiraide et al. [43]
				Kasemkijwattana et al. [44]

bFGF	Meniscal fibrochondrocytes	PGA scaffold culture	Enhance proliferation	Stewart et al. [45]

bFGF	Meniscal fibrochondrocytes	Alginate scaffold culture	Enhance proliferation and SMA expression	Cucchiarini et al. [46]

bFGF	BMSCs	Monolayer culture	Enhance proliferation	Sotiropoulou et al. [47]

bFGF	MSCs	Monolayer culture	Maintain the multilineage differentiation potential of MSCs	Buckley and Kelly [48]—
				Martin et al. [49]

bFGF and hypoxia	Meniscal fibrochondrocytes	Three-dimensional pellet culture	Reexpress collagen type II and PGs gene	Adesida et al. [50]

bFGF	Intervertebral disc cells	Monolayer and alginate beads culture	Suppress proteoglycan production	Li et al. [51]

bFGF	Articular chondrocytes	Monolayer culture	Suppress collagen type II and decorin synthesis	Sonal [52]

bFGF	Articular chondrocytes	Monolayer culture	Upregulate MMPs, aggrecanases, nitric oxide and superoxide anion expression	Muddasani et al. [53]

bFGF	Articular chondrocytes	Alginate culture	Antagonizes proteoglycan synthesis	Loeser et al. [54]

bFGF	Articular chondrocytes	Gelatin-chondroitin-hyaluronan hybrid scaffold culture	Repair with hyaline-like cartilage	Deng et al. [55]

bFGF and hypoxia	Meniscal fibrochondrocytes	PLLA scaffold culture	Enhance GAGs production and compressive properties of constructs	Gunja and Athanasiou [56]

bFGF and TGF-*β*3	None	Electrospun PCL scaffolds culture	Improve meniscus repair and scaffold integration	Ionescu et al. [57]

IGF–1	Meniscal fibrochondrocytes	Alginate scaffold culture	Increase collagen and GAG synthesis as well as mechanical properties	Puetzer et al. [58]

IGF-1	Meniscal fibrochondrocytes	Monolayer culture	Enhanced proliferation and ECM formation	Tumia and Johnstone [59]

IGF-1	BMSCs	Monolayer culture	Modulate chondrogenic differentiation	Longobardi et al. [60]

IGF-1	Meniscal fibrochondrocytes	Explant culture	Stimulated cell migration	Bhargava et al. [61]

IGF-1	BMSC (transfection of hIGF-1 gene)	Intra-articular injection in goat	Promote the repair of full-thickness meniscal defects	Zhang et al. [62]

IGF-1	Articular chondrocytes (transfection of hIGF-1 gene)	Polymerized fibrinogen in equine model	Enhance cartilage healing	Goodrich et al. [63]

IGF-1 and TGF-*β*1	BMSCs	Three-dimensional fibrin disk culture	Enhance chondrogenic differentiation	Worster et al. [64]

IGF-1 and TGF-*β*1	None	Explant culture	Improve repair of meniscus avascular zone	Izal et al. [65]

IGF–1, TGF-*β*1, bFGF	Fibroblast-like synoviocytes	PGA/PLLA scaffold culture	Enhance collagen type II and aggrecans expression	Fox et al. [66]

IGF and BMP-7	Articular chondrocytes	Monolayer culture	Suppress MMP-13 expression	Im et al. [67]

VEGF	None	VEGF-coated sutures in a sheep	Fail to promote meniscus healing	Petersen et al. [68]

HGF	Meniscal fibrochondrocytes (transfection of HGF-1 gene)	PGA scaffold in mice	Induce blood vessel formation in engineered constructs	Hidaka et al. [69]

CTGF	Meniscal fibrochondrocytes	Fibrin glue in rabbits model	Promote healing of meniscal defect in the avascular zone	He et al. [70]

CTGF	None	Hydrogel collagen scaffold in rats model	Enhance articular cartilage regeneration	Nishida et al. [71]

PDGF-AB	Meniscal fibrochondrocytes	Monolayer culture	Increase proliferation and matrix formation	Tumia and Johnstone [72]

PRP	Meniscal fibrochondrocytes	Gelatin hydrogel in a rabbit model	Promote meniscus repair	Ishida et al. [73]

## Abbreviations

GF: Growth factor
ECM: Extracellular matrix
GAG: Glycosaminoglycan
TGF-*β*: Transforming growth factor-*β*
FGF: Fibroblast growth factor
bFGF: Basic fibroblast growth factor
IGF-1: Insulin-like growth factor-1
CTGF: Connective tissue growth factor
VEGF: Vascular endothelial growth factor
PDGF: Platelet-derived growth factor
HGF: Hepatocyte growth factor
PRP: Platelet-rich plasma
BMP: Bone morphogenetic protein
OP-1: Osteogenic protein-1
EGF: Epidermal growth factor
NGF: Nerve growth factor
HIF-1: Hypoxia inducible factor-1*α*
C-ABC: Chondroitinase ABC
*α*-SMA: Alpha-smooth muscle actin
3D: Three-dimensional
MMP: Matrix metalloprotease
TIMP: Inhibitors of matrix metalloprotease
IL: Interleukin
TNF: Tumor necrosis factor
OA: Osteoarthritis
MSC: Mesenchymal stem cell
BMSC: Bone marrow stromal cell
hMSC: Human mesenchymal stem cell
CDMP: Cartilage-derived morphogenetic protein
PLLA: Poly L-lactic acid
PCL: Polycaprolactone
PGA: Polyglycolic acid
HSV: Herpes simplex virus
rAAV: Recombinant adeno-associated virus
KGN: Kartogenin
FDA: Food and Drug Administration.
